# Vitamin D Toxicity

**DOI:** 10.1590/2175-8239-JBN-2019-0192

**Published:** 2020-04-03

**Authors:** Kenneth Lim, Ravi Thadhani

**Affiliations:** 1 Harvard Medical School, Massachusetts General Hospital, Division of Nephrology, Department of Medicine, Boston, MA, EUA; 2 Massachusetts General Hospital, Partners Healthcare, Boston, MA, EUA

**Keywords:** Vitamin D, Poisoning, Toxicity, Hypercalcemia, Acute Kidney Injury, Vitamina D, Envenenamento, Toxicidade, Hipercalcemia, Lesão Renal Aguda

## Abstract

Fortification of food products with vitamin D was central to the eradication of rickets in the early parts of the 20th century in the United States. In the subsequent almost 100 years since, accumulating evidence has linked vitamin D deficiency to a variety of outcomes, and this has paralleled greater public interest and awareness of the health benefits of vitamin D. Supplements containing vitamin D are now widely available in both industrialized and developing countries, and many are in the form of unregulated formulations sold to the public with little guidance for safe administration. Together, this has contributed to a transition whereby a dramatic global increase in cases of vitamin D toxicity has been reported. Clinicians are now faced with the challenge of managing this condition that can present on a spectrum from asymptomatic to acute life-threatening complications. This article considers contemporary data on vitamin D toxicity, and diagnostic and management strategies relevant to clinical practice.

## Resumo

A suplementação de produtos alimentares com vitamina D foi fundamental para a erradicação do raquitismo no início do século XX nos Estados Unidos. Nos quase 100 anos subsequentes, o acúmulo de evidências vinculou a deficiência de vitamina D a uma variedade de desfechos, e isso tem levantado grande interesse público e conscientização dos benefícios à saúde da vitamina D. Os suplementos que contêm vitamina D estão agora amplamente disponíveis tanto nos países desenvolvidos quanto naqueles em desenvolvimento, e muitos estão na forma de formulações não regulamentadas, vendidas ao público com poucas orientações para uma administração segura. Juntos, isso contribuiu para uma transição na qual um aumento global dramático nos casos de toxicidade da vitamina D tem sido relatado. Médicos agora enfrentam o desafio de tratar essa condição que pode apresentar um espectro de complicações assintomáticas a agudas, com risco de vida. Este artigo considera dados atualizados sobre a toxicidade da vitamina D e estratégias de diagnóstico e manejo relevantes para a prática clínica.

## The other side of vitamin d therapy

An estimated 1 billion people worldwide are deficient or insufficient in vitamin D.^1^ This staggering statistic is worrying given that vitamin D is indispensable to human health. In fact, mounting experimental, observational, and epidemiologic evidence has linked low vitamin D levels to a number of adverse health outcomes, such as all-cause mortality, cardiovascular disease, reduced bone density, fracture risk, metabolic syndrome, malignancy, autoimmune conditions, and infection.^2^ Additionally, some evidence suggests that vitamin D status is a biomarker of lifestyle, given that unhealthy and sedentary lifestyles are associated with vitamin D insufficiency or deficiency, which itself represents a risk factor for adverse health outcomes.^3^ Lack of adequate sunlight exposure, skin color, and socio-religious practices are all contributors to vitamin D insufficiency or deficiency.

Given the high prevalence of vitamin D deficiency and the discovery of the link between vitamin D deficiency and the rickets epidemic at the dawn of the 20^th^ century, a public health campaign for sensible sun exposure started. By the 1930s, health authorities in the United States took action and introduced fortification of foods such as milk to improve vitamin D intake and status in the general population.^4^ This represented a major step toward the eradication of rickets. Since then, public interest in vitamin D supplementation grew as new links between its deficiency and various health outcomes emerged. Additionally, supplements containing vitamin D have become increasingly available in both industrialized and developing countries. This and the lack of proper guidance for therapeutic supplementation or public education, have likely contributed to complications on the other spectrum of abnormalities in vitamin D metabolism, namely vitamin D toxicity or hypervitaminosis D.

Analysis of temporal trends have revealed a steady uptrend in serum vitamin D levels, at least in several countries such as Ireland (between 1993-2013), Norway (between 1994-2008), and Canada (between 1995-1997 to 2005-2007).^5^ These increases have been attributed to a rise in the intake of food fortified with vitamin D, predominantly milk, and an increase in the use of over-the-counter supplements. These data coincide with an increase in the testing of vitamin D levels that has been reported by individual institutions around the developed world such as in the United States (San Francisco, from data captured between 2006-2009), England (London, between 2007-2010), Scotland (Glasgow, between 2008-2010), Canada (Ontario, from 2004-2010), and Australia (from, 2001-2010).^5^ Furthermore, in one retrospective analysis of data from the National Poison Data System (NPDS), toxic exposure to vitamin D increased from a mean of 196 cases per year from 2000 to 2005, to a staggering mean of 4535 exposures per year from 2005-2011.^6^ Accumulating reports of vitamin D toxicity suggest a transition point whereby clinicians are now faced with an increasing likelihood of having to manage patients with vitamin D intoxication that can have devastating health implications. An illustration is provided in the case vignette below. This review considers contemporary data on vitamin D toxicity, and diagnostic and management strategies relevant to clinical practice. The reader is referred elsewhere for a discussion on vitamin D physiology, metabolism, and current randomized trial data.^2^

## Case vignette

A 72-year-old male with a history of non-insulin dependent diabetes, deep vein thrombosis, and obstructive sleep apnea presented to the emergency room with altered mental status (AMS) and acute hypoxemic respiratory failure. The patient was found down by his son at his home for an unknown time period. Collateral history obtained from the son revealed that the patient was last seen in his usual state of health approximately 10 days prior to presentation.

On arrival to the ED, the patient was non-verbal and obtunded. He was tachycardic to the 100s beats/minute with an irregular rhythm, blood pressure 80s/60s mmHg, respiratory rate 18 beats/minute and a GCS of 8. In the ED, the patient was intubated for airway protection, given 3L NS, and started empirically on ceftriaxone, vancomycin, and acyclovir. Physical exam was remarkable for an obtunded patient, otherwise all other systems were largely negative. Laboratory investigations on presentation revealed a Cr elevated to 8.23 mg/dL, blood urea nitrogen (BUN) 158, Na 148 mmol/L, K+ 3.9 mmol/L, HCO_3_ 28 mmol/L, glucose 8.2 mg/dL, anion gap 24 mmol/L, lactate 3.2 mmol/L, pH 7.39, pCO_2_ 52, uric acid 14.0 mmol/L; uncorrected calcium 14.3 mmol/L, albumin 2.9 g/dL, PTH 7 pg/mL. Liver enzymes were within normal limits. Urinalysis revealed 1+ protein, 1+ glucose, no casts or dysmorphic cells. Head CT revealed no acute intracranial processes, chest x-ray was clear, and renal ultrasound showed a right kidney 12.4 cm, left kidney 11.9 cm and no hydronephrosis. Further work-up revealed no abnormalities on serum protein electrophoresis (SPEP), urine protein electrophoresis (UPEP), or serum free light chain analysis. PTHrP was 0.4 pmol/L, antineutrophil cytoplastic antibodies (ANCA) negative, C3 and C4 levels were within normal limits. Assessment of vitamin D revealed a markedly elevated 25(OH)D level >120 nmol/L and active 1,25(OH)_2_D of 39 pg/mL.

The patient was subsequently transferred to the medical ICU and developed anuria by day 1 of admission. He was started on intermittent hemodialysis by day 2 of admission. By day 3, his mental status improved and was subsequently extubated. By day 4, the patient was taken off hemodialysis and over the course of subsequent weeks made a complete recovery. With improving mental status, the patient reported that he had been consuming excessive amounts of dietary vitamin D that included cholecalciferol 30,000 - 50,000 IU daily in addition to taking a combined pill containing calcium 500 mg and ergocalciferol 400 IU daily.

## Causes of vitamin d toxicity

**Figure 1:**
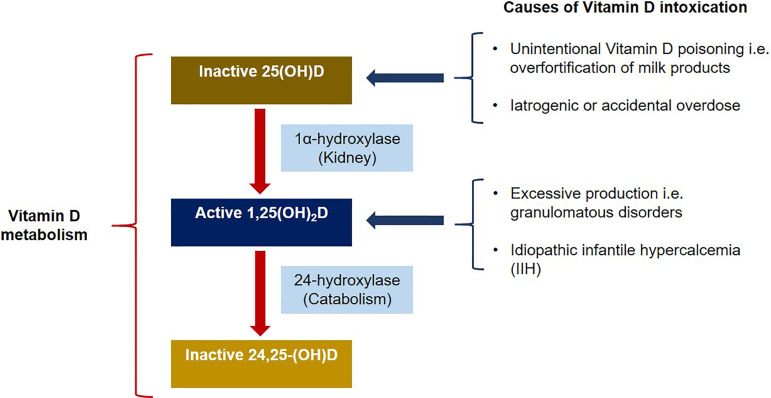
Causes and metabolism of vitamin D intoxication: Increased intake of vitamin D by causes such as vitamin D poisoning, iatrogenic, or accidental overdose raises the levels of serum inactive 25(OH)D. Inactive 25(OH)D (25-hydroxyvitamin D) is then converted to active 1,25(OH)2D (1,25-dihydroxyvitamin D) by 1α-hydroxylase in the kidney. Vitamin D toxicity can also occur via excessive production of active 1,25(OH)2D by causes such as granulomatous disorders or idiopathic infantile hypercalcemia (IIH). 1,25(OH)2D has a high affinity for vitamin D receptors (VDRs) resulting in increased gene expression of target cells. Catabolism of 1,25(OH)2D by 24-hydroxylase generates inactive 24,25-(OH)D (24-hydroxylase).

Vitamin D toxicity is usually iatrogenic or due to accidental overdose (Figure 1). Supplements containing vitamin D are now easily obtainable over-the-counter in pharmacies, grocery and food markets, and other retail stores. Some of these supplements exists as unregulated or unlicensed formulations.^7^ These factors together with the lack of public education for safe dosing has likely contributed to increasing reported cases of vitamin D toxicity. Unintentional vitamin D poisoning has also been associated with overfortification of milk^8^, poisoning by table sugar^9^, and contamination of cooking oil. In one report of hypervitaminosis D that occurred in eight patients, the source was found to be an error at a local dairy where excessive vitamin D fortification of milk up to 232,565 IU per quart instead of the standard 400 IU per quart occurred.^10^ Additionally, sadly, at times many cases of vitamin D intoxication result from doses of vitamin D prescribed far above the permissible limits.

In Kashmir, India, elderly patients have been given oral or injectable forms of various vitamins for minor ailments, generalized debility, or at times as a “wonder drug” according to one report.^11^ One case series reported 62 patients with malpractice-related vitamin D intoxication secondary to multiple injections of vitamin D (600,000 IU/injection), who presented with hypercalcemia and acute kidney injury (AKI).^11^ Joshi et al. reported 7 children in India who had received mal-practice related high doses of vitamin D injections (900,000-400,000,000 IU) for failure to thrive.^12^ In Brazil, one report describes an exponential increase in vitamin D intake over the past decade, and its indiscriminate use and manipulations in preparations may potentially enhance the incidence of vitamin D toxicity in that country.^13^ Additionally, cases of vitamin D toxicity have been reported after misunderstanding of physician instructions.^14^

Vitamin D toxicity can also occur secondary to excessive production of the active form of vitamin D, 1,25(OH)_2_D (1,25-dihydroxyvitamin D) in patients with granulomatous disorders such as sarcoidosis, tuberculosis, leprosy, fungal diseases, infantile subcutaneous fat necrosis, giant cell polymyositis, and berylliosis.^15^ High vitamin D levels in these conditions are related to abnormal extrarenal synthesis of 1,25(OH)_2_D. In lymphomas, the etiology of elevated vitamin D levels is still not fully understood and has been associated with paracrine regulation of tumor-associated macrophages.^16^ In rare cases, hypervitaminosis D occurs in idiopathic infantile hypercalcemia (IIH) due to loss-of-function mutation in the CYP24A1 gene, which encodes the vitamin D-metabolizing enzyme 24-hydroxylase.^17^

### Pathophysiology of vitamin d toxicity

The Institute of Medicine (IOM) Report in 2011 highlighted the upper limits for vitamin D intake on the basis of the effects of acute, short-term administration of high-dose vitamin D preparations and those that can occur secondary to chronic administration over years of supplementation.^18^ Acute vitamin D toxicity is usually caused by doses of vitamin D above 10,000 IU/day resulting in serum 25(OH)D concentrations >150ng/mL. Chronic vitamin D toxicity can potentially occur with administration of doses above 4,000 IU/day for extended periods, likely in the region of years resulting in 25(OH)D concentrations in the 50-150 ng/mL range.

Vitamin D intoxication occurs as a result of a vitamin D metabolite reaching the vitamin D receptor (VDR) in the nucleus of target cells and causing exaggerated gene expression.^19^ Three hypotheses to explain vitamin D toxicity have been previously put forward:

- Increased vitamin D intake increases serum concentrations of the active form of vitamin D, 1,25(OH)_2_D (1,25-dihydroxyvitamin D).^19^ 1,25(OH)_2_D has a high affinity for VDRs and is a critical ligand with access to transcriptional signal transduction machinery in the cell.^20^
- Vitamin D intake increases inactive plasma 25(OH)D (25-hydroxyvitamin D) and saturates the binding capacity of vitamin D binding protein (VDBP).^19^ 25(OH)D at higher concentrations has a greater affinity for VDRs (in a dose-dependent effect) compared to other vitamin D metabolites, that enter cells where it has direct effects on gene expression.^20^
- Increased vitamin D intake raises the concentrations of many vitamin D metabolites, particularly 25(OH)D.^19^ In states of hypervitaminosis D, the concentrations of various vitamin D metabolites, including 25(OH)D, 24,25(OH)_2_D, 25,26(OH)_2_D, and 25(OH)D-26,23-lactone, increase significantly.^21^ These metabolites exceed the VDBP capacity and cause the release of “free” 1,25(OH)_2_D, which enter target cells by diffusion and subsequently stimulates the VDR.

### Clinical features and diagnosis of vitamin d toxicity

The presentation of vitamin D toxicity can range from asymptomatic to severe neuropsychiatric and life-threatening features. Vitamin D toxicity is largely characterized by severe hypercalcemia that may persist for a prolonged time. Clinical manifestations of vitamin D toxicity are varied, but largely related to hypercalcemia and include neuropsychiatric (such as confusion, psychosis, stupor, or coma), gastrointestinal (abdominal pain, vomiting, polydipsia, anorexia, constipation, pancreatitis), cardiovascular (hypertension, shortened QT interval, ST segment elevation, bradyarrhythmias, first degree heart block), and renal (hypercalciuria, acute kidney injury (AKI), dehydration and nephrocalcinosis) complications.^15^ Additional complications of hypercalcemia include band keratopathy, hearing loss, and painful periarticular calcinosis. In animal studies, hypervitaminosis D has been shown to cause widespread vascular calcification.^22^

Serum inactive 25(OH)D levels >100 ng/mL (250 nmol/L) have been defined as hypervitaminosis D, whereas serum levels >150 ng/mL (375 nmol/L) have been proposed to define vitamin D intoxication by the Endocrine Society.(14) Other laboratory findings include hypercalcemia, hypercalciuria, and very low or undetectable parathyroid hormone (PTH) levels. The 1,25(OH)_2_D concentration, which is the active form of vitamin D, may be within the reference range, slightly increased or reduced. The latter finding is secondary to inhibition of 1a-hydroxylase activity responsible for synthesizing active 1,25(OH)_2_D and enhancement of 24-hydroxylase activity involved in its catabolic pathway.^23^ Exogenous administration of active vitamin D metabolite or increased endogenous production can result in elevated 1,25(OH)_2_D concentrations, and normal or decreased 25(OH)D levels.

It should be noted that serum 1,25(OH)_2_D has been reported to be falsely elevated in patients with vitamin D intoxication with certain laboratory assays, particularly radioimmunoassay due to significant cross-reactivity of very high levels of 25(OH)D.^24^ Additionally, circulating levels of 25(OH)D may not always reflect its true values and activities, in part due to many extrarenal tissues expressing signaling components for vitamin D including the 1a-hydroxylase enzyme that is responsible for synthesizing biologically active 1,25(OH)_2_D. For example, vascular smooth muscle cells (VSMCs) express a functional VDR and the 1a-hydroxylase enzyme, enabling arteries to synthesize bioactive vitamin D and establish an autocrine/paracrine hormonal system to regulate cardiovascular health locally.^2^^,^ 25. This suggests that the endocrine, autocrine, and paracrine functions of vitamin D may not always be reflected from its serum 25(OH)D levels.^26^ Additionally, circulating 25(OH)D concentrations are influenced by many factors such as race, pigmentation, age, season, latitude, weather conditions, dietary habits, and exposure to sunlight. Many peripheral tissues are able to convert circulating 25(OH)D to active 1,25(OH)_2_D to meet local requirements and this may not be reflected by its serum levels.^26^

### Management of vitamin d toxicity

The main goal of treatment during vitamin D toxicity is emergent resuscitation in an unstable patient and correction of hypercalcemia. Hypercalcemia due to vitamin D overdose theoretically can last up to 18 months following discontinuation of administration. This is due to the slow release of stored vitamin D from fat deposits. Therefore, sustaining normocalcemia is just as pivotal as acute treatment of hypercalcemia. Additionally, vitamin D_2_ or D_3_ has a high lipid solubility in liver, muscles, and fat tissues and a long half-life in the body. However, 25(OH)D and 1,25(OH)_2_D have shorter half-lives at 15 days and 15 hours, respectively. Therefore, overdose of 25(OH)D can persist for weeks.

**Figure 2:**
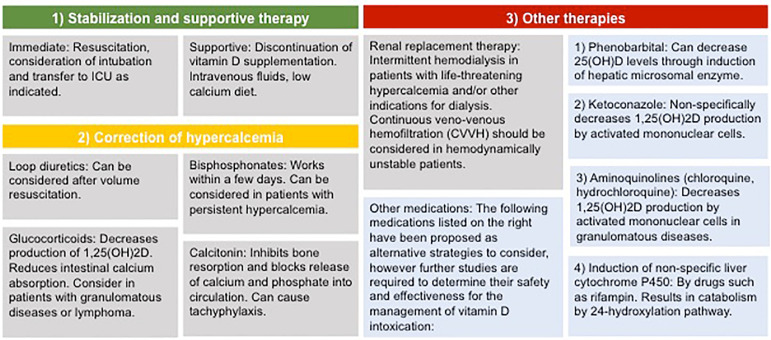
Management of vitamin D intoxication: The management of vitamin D toxicity can be categorized into three steps: 1) Stabilization and supportive therapy. In unstable patients, immediate resuscitative measures should be undertaken including intubation, administration of intravenous fluids, and transfer to an intensive care unit (ICU) as appropriate; 2) Correction of hypercalcemia takes advantage of several different classes of medications, including loop diuretics, bisphosphonates, glucocorticoids or calcitonin; 3) Other therapies to consider most commonly include renal replacement therapy (RRT) in severe cases.

Therapeutic strategies for vitamin D toxicity can be categorized into 1) stabilization and supportive treatment; 2) correction of hypercalcemia, and 3) other therapies to reduce vitamin D levels as illustrated in Figure 2.

### Adequate concentrations and dosing of vitamin D

Vitamin D status is determined by measuring 25(OH)D concentrations. Unfortunately, there remains controversy as to the definitions of vitamin D deficiency, sufficiency, and recommended dietary allowance (RDA) to help guide optimal supplementation.^18^ In the 2011 dietary reference intake (DRI) report by the IOM, bone health was reported to be the only outcome whereby causality has been established by available evidence. The evidence for other extraskeletal chronic disease outcomes was deemed to be inconsistent or inconclusive to establish causality and insufficient to serve as a basis for DRI. A 25(OH)D level of 16 ng/mL (40 nmol/L) has been suggested by the 2011 IOM report to meet the needs of approximately half the population (median population requirement) and levels of 20 ng/mL (50 nmol/L) meet the needs of at least 97.5% of the population.^18^ However, the UK Department of Health and Scientific Advisory Committee on Nutrition (SACN) define vitamin D deficiency as <25 nmol/L (10 ng/mL). Additionally, others have argued that the deficiency threshold should be significantly higher, at 50 nmol/L (20 ng/dL).^1^

The IOM recommends an RDA for vitamin D of 600 IU/day for individuals aged 1 to 70 years and 800 IU/day for those aged over 70 years. These recommendations are based on the averages of serum 25(OH)D concentrations pooled from 32 studies. This RDA is expected to achieve a 25(OH)D level of 50 nmol/L or more in 97.5% of healthy individuals per the IOM.^27^ Further trials are critically needed to help establish vitamin D thresholds, refine the RDA based on population and individual characteristics, and reduce the uncertainty over the benefits of vitamin D supplementation. Additionally, there is increasing recognition that extraskeletal outcomes should be taken into account for defining sufficiency thresholds.

## Conclusions

Both underdosing and overtreatment with vitamin D can have considerable health consequences. Vitamin D toxicity remains an ongoing issue and its incidence is likely to continue increasing, owing to the widespread availability of over-the-counter preparations and public interest.^7^ Measures to help prevent and manage cases of vitamin D excess are critically needed and is of substantial public health importance. We suggest the following considerations in regard to the prevention of vitamin D toxicity:

1. Clear physician-patient communication should be emphasized when prescribing vitamin D-containing formulations and the risks associated with excess administration should be communicated.
2. Health care providers and community pharmacists should be aware of the various vitamin D preparations, their variability, safety profile, and risks associated with supratherapeutic dosing to help in clinical-decision making and provision of appropriate recommendations. Appropriate education of health professionals will assist in preventing inappropriate prescribing or dispensing.
3. High dose vitamin D administration should be avoided until serum 25(OH)D and calcium levels have been assessed. This will help assess the need for high dose vitamin D and avoid potential toxicity by empiric treatment.
4. One case report described that a single dose of 2,000,000 IU of vitamin D was given in error to two nursing home residents.^28^ This suggests the need to regulate the availability of multiple use bottles with more conventional dose formulations in line with current IOM and other published guidelines.^7^
5. Consideration of vitamin D toxicity or excess should be made in patients presenting with hypercalcemia or hypercalciuria.

## References

[1] Holick MF (2007). Vitamin D deficiency. N Engl J Med.

[2] Hiemstra T, Lim K, Thadhani R, Manson JE (2019). Vitamin D and Atherosclerotic Cardiovascular Disease. J Clin Endocrinol Metab.

[3] Gunta SS, Thadhani RI, Mak RH (2013). The effect of vitamin D status on risk factors for cardiovascular disease. Nat Rev Nephrol.

[4] Reichrath J (2014). Solar ultraviolet radiation, vitamin D and skin cancer surveillance in organ transplant recipients (OTRs): an update. Adv Exp Med Biol.

[5] Sharma LK, Dutta D, Sharma N, Gadpayle AK (2017). The increasing problem of subclinical and overt hypervitaminosis D in India: An institutional experience and review. Nutrition.

[6] Spiller HA, Good TF, Spiller NE, Aleguas A (2016). Vitamin D exposures reported to US poison centers 2000-2014: Temporal trends and outcomes. Hum Exp Toxicol.

[7] Taylor PN, Davies JS (2018). A review of the growing risk of vitamin D toxicity from inappropriate practice. Br J Clin Pharmacol.

[8] Chiricone D, De Santo NG, Cirillo M (2003). Unusual cases of chronic intoxication by vitamin D. J Nephrol.

[9] Vieth R, Pinto TR, Reen BS, Wong MM (2002). Vitamin D poisoning by table sugar. Lancet.

[10] Jacobus CH, Holick MF, Shao Q, Chen TC, Holm IA, Kolodny JM (1992). Hypervitaminosis D associated with drinking milk. N Engl J Med.

[11] Wani M, Wani I, Banday K, Ashraf M (2016). The other side of vitamin D therapy: a case series of acute kidney injury due to malpractice-related vitamin D intoxication. Clin Nephrol.

[12] Joshi R (2009). Hypercalcemia due to hypervitaminosis D: report of seven patients. J Trop Pediatr.

[13] Guerra V, Vieira OM, Laurindo AF, Paula FJ, Moyses M (2016). Hypercalcemia and renal function impairment associated with vitamin D toxicit y: case report. J Bras Nefrol.

[14] Vogiatzi MG, Jacobson-Dickman E, DeBoer MD, Drugs, Therapeutics Committee of The Pediatric Endocrine S (2014). Vitamin D supplementation and risk of toxicity in pediatrics: a review of current literature. J Clin Endocrinol Metab.

[15] Marcinowska-Suchowierska E, Kupisz-Urbanska M, Lukaszkiewicz J, Pludowski P, Jones G (2018). Vitamin D Toxicity-A Clinical Perspective. Front Endocrinol (Lausanne).

[16] Hewison M, Kantorovich V, Liker HR, Van Herle AJ, Cohan P, Zehnder D (2003). Vitamin D-mediated hypercalcemia in lymphoma: evidence for hormone production by tumor-adjacent macrophages. J Bone Miner Res.

[17] Tray KA, Laut J, Saidi A (2015). Idiopathic Infantile Hypercalcemia, Presenting in Adulthood--No Longer Idiopathic Nor Infantile: Two Case Reports and Review. Conn Med.

[18] Ross AC, Manson JE, Abrams SA, Aloia JF, Brannon PM, Clinton SK (2011). The 2011 report on dietary reference intakes for calcium and vitamin D from the Institute of Medicine: what clinicians need to know. J Clin Endocrinol Metab.

[19] Jones G (2008). Pharmacokinetics of vitamin D toxicity. Am J Clin Nutr.

[20] Bikle DD, Gee E, Halloran B, Kowalski MA, Ryzen E, Haddad JG (1986). Assessment of the free fraction of 25-hydroxyvitamin D in serum and its regulation by albumin and the vitamin D-binding protein. J Clin Endocrinol Metab.

[21] Pettifor JM, Bikle DD, Cavaleros M, Zachen D, Kamdar MC, Ross FP (1995). Serum levels of free 1,25-dihydroxyvitamin D in vitamin D toxicity. Ann Intern Med.

[22] Wang J, Zhou JJ, Robertson GR, Lee VW (2018). Vitamin D in Vascular Calcification: A Double-Edged Sword?. Nutrients.

[23] Tebben PJ, Singh RJ, Kumar R (2016). Vitamin D-Mediated Hypercalcemia: Mechanisms, Diagnosis, and Treatment. Endocr Rev.

[24] Hawkes CP, Schnellbacher S, Singh RJ, Levine MA (2015). 25-Hydroxyvitamin D Can Interfere With a Common Assay for 1,25-Dihydroxyvitamin D in Vitamin D Intoxication. J Clin Endocrinol Metab.

[25] Zehnder D, Bland R, Williams MC, McNinch RW, Howie AJ, Stewart PM (2001). Extrarenal expression of 25-hydroxyvitamin d(3)-1 alpha-hydroxylase. J Clin Endocrinol Metab.

[26] Razzaque MS (2018). Can adverse effects of excessive vitamin D supplementation occur without developing hypervitaminosis D?. J Steroid Biochem Mol Biol.

[27] Food and Nutrition Board IoM (2011). Dietary Reference Intakes for Calcium and Vitamin D.

[28] van den Ouweland J, Fleuren H, Drabbe M, Vollaard H (2014). Pharmacokinetics and safety issues of an accidental overdose of 2,000,000 IU of vitamin D3 in two nursing home patients: a case report. BMC Pharmacol Toxicol.

